# Secondary Endoleak Management Following TEVAR and EVAR

**DOI:** 10.1007/s00270-020-02572-9

**Published:** 2020-08-10

**Authors:** Seyed Ameli-Renani, Vyzantios Pavlidis, Robert A. Morgan

**Affiliations:** 1 Department of Radiology, St George’s University Hospitals NHS Foundation Trust, London, UK; 2 Vascular & Cardiac Surgery Research Centre, St George’s University of London, Cranmer Terrace, London, SW17 ORE UK

## Abstract

Endovascular abdominal and thoracic aortic aneurysm repair and are widely used to treat increasingly complex aneurysms. Secondary endoleaks, defined as those detected more than 30 days after the procedure and after previous negative imaging, remain a challenge for aortic specialists, conferring a need for long-term surveillance and reintervention. Endoleaks are classified on the basis of their anatomic site and aetiology. Type 1 and type 2 endoleaks (EL1 and EL2) are the most common endoleaks necessitating intervention. The management of these requires an understanding of their mechanics, and the risk of sac enlargement and rupture due to increased sac pressure. Endovascular techniques are the main treatment approach to manage secondary endoleaks. However, surgery should be considered where endovascular treatments fail to arrest aneurysm growth. This chapter reviews the aetiology, significance, management strategy and techniques for different endoleak types.

## Fact Sheet

### A: Ten Most Important Points Regarding Secondary Endoleak Management Following TEVAR and EVAR

1. CTA is the main imaging investigation for assessing and characterising secondary endoleaks.
2. EL1 endoleaks are high pressure and require prompt treatment.
3. The main endovascular therapeutic options for EL1 include EndoAnchors, aortic cuffs and embolisation.
4. EL2 are low pressure, often benign and only warrant treatment if associated with a sac size increase of at least 5 mm.
5. An occult EL1 and EL3 should be considered and excluded when facing a suspected EL2 with increasing sac size.
6. Embolisation is the mainstay treatment for EL2 with increasing sac size
7. Techniques for catheterising the endoleak sac in EL2 include transarterial, transiliac paraendograft, direct sac puncture and transcaval embolisation.
8. Embolisation agents for both EL1 and EL2 embolisation include coils, and liquid embolics, including Onyx, glue and thrombin.
9. The outcomes of type 2 embolisation in experienced hands are very good if experts select the appropriate embolisation method for the specific patient anatomy and perform a technically complete embolisation.
10. Embolisation has a small but defined role in the management of endoleaks after TEVAR.

### B: Five Most Important Numbers with Respect to Endoleak Management After EVAR and TEVAR

11. The combined approach of DUS, CTA and MRI detects and characterises secondary endoleaks in 91% of cases.
12. Embolisation has a technical success above 95% and mid-term success of 80% for EVAR EL1a.
13. Embolisation of type 2 endoleaks is indicated for an increase in sac size of 5 mm on sequential imaging.
14. Regarding outcomes of embolisation for type 2 endoleaks, a recent review indicated that the technical success rate for direct sac puncture embolisation (81%) is higher than transarterial embolisation (63) and a lower rate of recurrence (19% vs 36%).
15. Type 1 endoleaks occur in 3.3–16% after TEVAR, and type 2 endoleaks occur in 3.3% of all TEVAR cases. Embolisation of left subclavian artery associated type 2 endoleaks has a technical and clinical success of 100%.

### C: Key References

- 1. Guo Q, Zhao J, Ma Y, Huang B, Yuan D, Yang Y, et al. A meta-analysis of translumbar embolization versus transarterial embolization for type II endoleak after endovascular repair of abdominal aortic aneurysm. J Vasc Surg [Internet]. 2019;1–7. Available from: https://doi.org/10.1016/j.jvs.2019.05.074
- 2. Ultee KHJ, Büttner S, Huurman R, Bastos Gonçalves F, Hoeks SE, Bramer WM, et al. Editor’s Choice – Systematic Review and Meta-Analysis of the Outcome of Treatment for Type II Endoleak Following Endovascular Aneurysm Repair. Eur J Vasc Endovasc Surg [Internet]. 2018 Dec [cited 2019 Dec 11];56(6):794–807. Available from: https://www.ncbi.nlm.nih.gov/pubmed/30104089
- 3. Ameli-Renani S, Pavlidis V, Morgan RA. Early and midterm outcomes after transcatheter embolization of type I endoleaks in 25 patients. J Vasc Surg. 2017;65(2).

### D: Two Messages About Endoleak Management Following TEVAR and EVAR

- 1. The primary therapy for type 1, 2 and 3 endoleaks after EVAR and TEVAR involves endovascular methods in the majority of cases. Many of the therapeutic options require the insertion of additional endografts in conjunction with additional endovascular methods, e.g. EndoAnchors, chimneys, etc.
- 2. Embolisation plays a key role in the treatment of type 2 endoleaks and EVAR and TEVAR. Embolisation plays a small, but significant role in the management of challenging type 1 endoleaks after EVAR and TEVAR if no other endovascular solution is feasible.

### E: Prediction for the Next Five Years

The therapeutic algorithm for all endoleaks will continue as described in this manuscript. It would be surprising to see any significant therapeutic advances or change in the approach to the management of any of the endoleak types. However, with increasing recognition that treating patients with hostile proximal necks by standard EVAR, and with the potential ramifications of planned UK NICE EVAR guidelines, we may see a significant reduction in the frequency of type 1a endoleaks after EVAR.

## Introduction

Endovascular abdominal and thoracic aortic aneurysm repair (EVAR) and (TEVAR) have become the mainstay of therapy of many pathologies of the abdominal and thoracic aorta. Moreover, the development of complex endograft technologies such as fenestrated EVAR (FEVAR), branched EVAR (BEVAR) and parallel grafts enables increasingly challenging anatomy to be treated. However, despite the increased number of procedures and diversity of techniques, the management of endoleaks remains a challenge for aortic specialists. Endoleaks (EL) may compromise long-term endograft viability and some are associated with an increased risk of rupture, thereby necessitating long-term surveillance and secondary interventions. Thus, early detection and classification of endoleaks is crucial for optimal management planning. Endoleaks may be classified as primary or secondary endoleaks. Primary endoleaks appear within 30 days post-procedure and secondary (or late) endoleaks are detected more than 30 days after the procedure and after previous negative imaging. Endoleaks are also classified on the basis of their anatomic site and aetiology and are subdivided into five types (Table 1) [1]. Type 1 endoleak (EL1) is caused by inadequate apposition of the endograft to the vessel wall (attachment site) and is subclassified as EL 1a for proximal endoleak, EL 1b for distal attachment site endoleak and EL Ic for lack of seal by an iliac occlude plug in aorto-uni-iliac repair with a crossover graft; type 2 endoleak (EL2) involves perfusion of the aneurysm sac from collateral vessels; type 3 endoleak (EL3) describes stent graft component separation or endoleak due to a fabric tear; type 4 endoleak (EL4) represents an endoleak due to porosity of the graft; and type 5 endoleak (EL5), also known as endotension, is present when there is expansion of the sac without an apparent endoleak on imaging. With developments in endograft fabric technology, type 4 endoleaks are of historical value and will not be further described. A wide range of treatment options exist including transarterial embolisation, percutaneous direct sac puncture embolisation, transcaval embolisation, surgical and conservative management. The criteria for best management should be tailored to each individual patient after careful planning and multidisciplinary team discussion.

**Table 1:** Summary of classification of endoleaks and their management

Endoleak	Cause of sac perfusion	Management
1	Flow from the proximal or distal graft attachment site	Prompt
a	Proximal graft attachment site	Angioplasty, Palmaz or cuff extension, chimney extension and embolisation
b	Distal graft attachment site	Angioplasty and extension of distal limb
c	Endoleak at the site of an iliac occluder plug	Insertion of an additional iliac occluder plug or embolisation
2	Retrograde flow through patent aortic side branch vessels	Conservative if sac size stable Embolisation if sac size increase
3	Mechanical graft failure	Prompt
a	Modular disconnection	Placement of additional endograft components
	Leak at a fenestration, branch or visceral stent	
b	Fabric tear	
4	Graft porosity	Conservative. Transient phenomenon
5	Sac size increase with no visible endoleak	May consider catheter angiography with cone beam CT

This article is focused on the diagnosis and management of secondary type 1, 2 and 3 endoleaks after EVAR and TEVAR.

## Diagnosis of Secondary Endoleaks

Numerous imaging modalities are available to detect and characterise endoleaks. However, factors such as the patient’s BMI, anatomy, endoleak type and size, local expertise and costs play a role in deciding optimal imaging follow-up protocols.

CT angiography (CTA) appears to be the gold standard for the diagnosis of both thoracic and abdominal endoleaks. The technique is probably optimal when a pre-contrast scan followed by an arterial and delayed phase study is performed, with endoleaks best appreciated on the delayed phase imaging [2, 3]. However, due to increase radiation exposure and cost considerations [4], usually an arterial phase or a dual bolus scan is sufficient to depict the endoleak. Our institution primarily employs a single arterial phase protocol for standard EVAR and TEVAR follow-up, with dual and triple phase imaging reserved for problem solving.

Doppler and contrast-enhanced ultrasound (CEUS) are commonly used in surveillance after EVAR, providing an accessible and affordable modality, with no radiation and high accuracy when performed by an experienced operator.

Similarly, contrast-enhanced MRI/MRA does not expose the patient to ionising radiation. In some patients, contrast-enhanced MRI appears to be superior to CTA to demonstrate occult endoleaks Nevertheless, MRI is not included in routine follow-up protocols due to the high costs, the prolonged examination time, the restricted availability and the common use of MR-incompatible endografts.

Conventional catheter angiography and/or C-Arm CTA (e.g. DynaCT—Siemens, Germany) is used as a problem-solving tool when an endoleak cannot be classified, or if there is a sac size increase without a visible endoleak on non-invasive imaging. In practice, catheter angiography is seldom positive in these latter cases.

The combined approach of DUS, CTA and MRI can raise the detection rate of endoleaks to 91% [5]. However, the necessary lifelong surveillance of this patient group increases the costs of aortic repair by 50% [4]. State-of-the-art imaging is crucial to guide optimal management for EVAR and TEVAR complications, especially endoleaks.

## Management of Secondary Endoleaks—EVAR

1. **Type 1 Endoleaks**

Type 1 endoleaks occur in up to 9% of cases [6] and is recognised as an indication for reintervention due to the high risk for rupture in up to 52% of cases [7, 8]. The risk of rupture is even higher (3.37%) when there is a combination of high-pressure endoleaks EL1 and EL3. Type 1a endoleaks are related to adverse proximal neck anatomy and there is an increased risk of these as more challenging aortic aneurysms are treated using endografts.. Secondary EL1 occur in 2.2 to 15% [9, 10]. Early EL1a is a common complication (30%) after snorkel/chimney EVAR technique, with high spontaneous resolution in up to 71.8% at 12 months and a low reintervention rate at 3.3% [11]. Late EL1a has been reported in up to 7% of patients after chimney EVAR [9].

Type 1b endoleak is caused by an inadequate seal at the distal landing zone. Late type 1b endoleaks are reported in 2.3% at a mean follow-up of 32.8 months [10]. Similar to EL1a, EL1b are associated with an increase in sac size and aneurysm rupture.

### Management of Type 1a Endoleak

Endovascular management of EL1a is mandatory, because of the documented risk of rupture. Technical success rates are high when managing intra-procedural or early EL1a, in the order of 90–100% [12–14]. Multiple options for reintervention are available, depending on the primary aortic repair technique that was used and the appearance on imaging. Standard techniques for primary endoleaks may include balloon dilatation and the insertion of giant balloon expandable stents in the neck to promote a proximal seal. However, these are seldom useful in secondary EL1a. Proximal endograft extension, either alone or in combination with chimney grafts, may be useful in selected patients with appropriate anatomy. More complex management options include FEVAR or BEVAR techniques. In addition, EndoAnchors (Medtronic, Santa Rosa, CA) provide proximal fixation of the endograft to the aortic wall with promising outcomes with technical success in 95%, residual EL1a in 9.1% and freedom from reintervention in 94.4% of patients treated [15].

If patients do not respond, are unsuitable, or are unfit for the above techniques, transcatheter embolisation is an alternative approach that can be used to manage the EL1a. Embolisation has an increased role for EL1a after ChEVAR because of the limited alternative options available.

Embolisation technique: The most common access for the embolisation is the common femoral artery, although alternative routes can be used such as the transradial [16] and transbrachial routes [17–19].

**Fig. 1:**
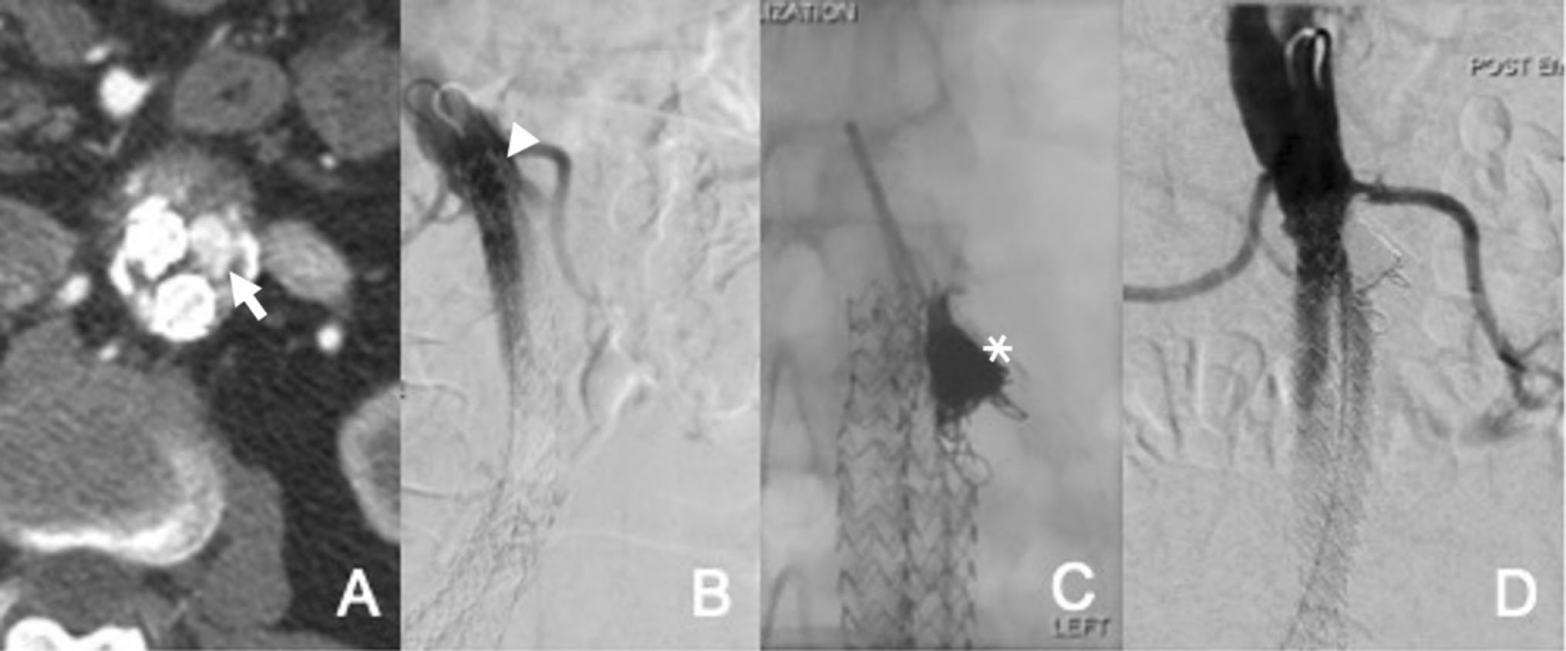
EL1a embolisation. **A** Axial CT angiogram shows proximal EL1 in patient with a Nellix endograft (arrow). **B** Aortic angiograms confirm EL1a (arrow head). **C** Embolisation of the endoleak cavity with coils and Onyx via a microcatheter following catheterisation of the endoleak cavity with reverse shaped catheter (asterix). **D** Final angiogram shows successful endoleak embolisation

The author’s preferred technique (Fig. 1) is through a retrograde femoral approach to advance a 45 cm long, 6F sheath (e.g. Destination, Terumo, Tokyo, Japan) to just below the top end of the endograft. An angiogram is performed from a flush catheter to depict the endoleak and to exclude additional other endoleak types. A reverse curve catheter (e.g. 5F Simmons; Cook Inc, Bloomington, Ind) with a hydrophilic wire is used to access the perigraft endoleak space. A dimethyl sulphoxide (DMSO) compatible microcatheter (e.g. 2.7F Progreat [Terumo Corp], Marathon or Echelon [Medtronic, Santa Rosa, Calif], or 2.95F PX SLIM [Penumbra, Alameda, Calif]) is advanced coaxially into the endoleak cavity. An endoleakogram is performed to better assess the size, geometry and the neck size and location of the endoleak nidus. The endoleakogram also enables the interventionist to evaluate for the presence of exit vessels, to find the best projection for the C-arm to visualise the endoleaks and to also create a road map for the embolisation procedure. Depending to the anatomy of the endoleak nidus, embolisation is performed using either ethylene vinyl alcohol copolymer (Onyx, Medtronic, Santa Rosa, Calif) alone or a combination of detachable coils (e.g. Ruby, Penumbra) and Onyx-34. Completion aortography is performed to assess for residual filling of the endoleak [20]. Complications are limited to puncture site access site hematomas and non-target embolisation with reflux of a small volume of the liquid embolic agent into the aorta, which is seldom clinically problematic.

Outcomes of embolisation: Embolisation is usually successful with immediate technical success (TS) approaching 100% in small series single-centre studies after EVAR (22 & 25 patients), EVAS (7 patients) and chimney (9 patients) techniques [21–25]. Reports indicate successful embolisation can be achieved using coils, Onyx or glue (*N*-butyl cyanoacrylate (NBCA)), or a combination [26–28] (Table 2).

**Table 2:** Main publications on outcomes of EL1 embolisation

Years	Authors	Pt	Treatment	Embolic Material	Technical success (%)	Mean follow-up (months)	Clinical success (%)
2018	Ierardi et al. [43]	8	Embolisation	Onyx and coils in 3, NBCA and Onyx in 1, Onyx and coils in 1	100 (8/8)	16.8	100 (8/8)
2018	Stenson et al. [44]	15	9 Embolisations 6 proximal extensions	Onyx + coils	100 9/9	36	–
2017	van de Ham et al. [45]	40	17 Embolisations 13 OC 10 Ch-EVAS	Onyx ± coils	96.5 (overall EL1 treatment)	1–6	–
2017	Marcelin et al. [23]	9	Embolisation	Onyx ± coils	100 9/9	15.9 ± 11.36	89 8/9
2017	Ameli-Renani et al. [21]	25	Embolisation	Onyx ± coils	100 (25/25)	10	80–85
2017	Graif et al. [46]	8	Embolisation 6 ELIa, 2 ELIb	Onyx ± coils	88 (7/8)	6.9	71(5/7)
2015	Gandini et al. [17]	1	Transcaval Embolisation	Cuff Zenith (Cook Medical, Bloomington, Ind) and thrombin and coils	100 (1/1)	12	100 (1/1)
2014	Eberhardt et al. [47]	8	Embolisation 6 ELIa, 1 ELIb, 1 ELIa & ELIb	Onyx ± coils	100 (8/8)	13.2 (8–24)	88 (7/8)
2013	Katada et al. [48]	1	Embolisation and cuff	Coils NBCA-lipiodol embolisation was performed (B), then the Zenith TX2 extension cuff (Cook Medical, Bloomington, Ind)	100 (1/1)	6	100 (1/1)
2011	Henrikson et al. [27]	6	Embolisation 5 ELIa, 1 ELIb	Onyx	100% (6/6)	3–18	100% (6/6)
2011	Choi et al. [49]	7	Embolisation and cuff	N-BCA ± coils	86% (6/7)	18 (0–53	86% (6/7)
2010	Lu et al. [50]	42	Embolisation 5 ELIa, 1 ELIb, 1 EL1a & ELIb	N-BCA or Onyx ± coils, fibrin glue injection	98% (41/42)	40	83% (35/42)
2010	Grisafi et al. [51]	1	Onyx	Embolisation device, Onyx (Micro Therapeutics Inc, Irvine, Calif)	100% (1/1)	12	100% (1/1)
2010	Loffroy et al. [52]	1	Transarterial microcoil Embolisation	Detachable microcoils into the nidus while an intra-aortic balloon catheter was inflated at the same time	100% (1/1)	6	100% (1/1)
2005	Golzarian et al. [53]	32	Embolisation 32 ELIa	Coils with or without gelatin sponge or thrombin	91% (29/32)	38.6	91% (20/22)
2003	Maldonandο et al. [54]	17	Embolisation 13 ELIa, 4ELIb	10 n-BCA, 3 coils, 4 cuff	94% (16/17)	6.9 (0–19)	88% (15/17)
1999	Amesur et al. [55]	5	Embolisation 1 ELIa, 4 ELIb	Coils	100% (5/5)	8 (3–17)	100% (5/5)

### Management of Type 1b Endoleaks

In general, these are easier to treat than EL1a endoleaks. Standard treatment consists of insertion of an additional endograft distally to achieve a distal seal. If there is insufficient space to extend to the origin of the internal iliac artery (IIA), then it is necessary to extend endograft coverage into the external iliac artery (EIA). The internal iliac artery can be overstented or embolised depending on whether there is a risk of a type 2 endoleak by covering the IIA (e.g. if the common iliac artery (CIA) is aneurysmal). Embolisation of the IIA can be performed with coils or plugs. In a two-centre study from 2018, 35 late 1b endoleaks that were treated by endograft extension demonstrated a 100% TS rate, 100% freedom from re-intervention at a mean follow-up at 20 months and no requirement for open surgical treatment [29]. Additional novel technologies that allow the preservation of the IIA include iliac branched devices and parallel grafts. Embolisation has a role in a very small minority of patients if no other option is available or feasible. However, in reality this situation is very rare after EVAR as distal endograft extension, with or without preservation of the IIA is feasible.

### Summary of Secondary Type 1 Endoleaks After EVAR

All current techniques available for managing EL1a, whether complex endovascular techniques such as FEVAR, BEVAR and ChEVAR, or simpler approaches such as EndoAnchors, embolisation techniques, cuff and/or Palmaz stent, demonstrate high technical and clinical success rates when used with the proper indication. Embolisation techniques, with success rates from 86 to 100%, should be considered when there is insufficient neck length for stent graft extension, when the other techniques have failed, or when the patient is unfit for more complex therapies. When primary treatment involved chimney EVAR, embolisation techniques can be used as the first line treatment for a persistent secondary type 1a endoleak.

2. **Type 2 Endoleaks**

Secondary type 2 endoleaks (EL2) are the most common endoleaks following EVAR and remain the main cause of repeat intervention [30]. They occur despite complete exclusion of the aneurysm at the proximal and distal attachment sites. Type 2 endoleaks are caused by retrograde blood flow into the sac from branches of the endograft-covered native aorta or iliac vessels. There is usually one dominant inflow artery, most commonly the IMA or a lumbar artery, and often one or more outflow arteries. Patency of the IMA and one or more lumbar arteries pre-EVAR, as well as larger aneurysms and aneurysms with significant thrombus burden in the sac have found to increase the risk of developing type 2 endoleaks [31, 32]. Below, we will address the best approach to management of secondary type 2 endoleaks.

### Indications for intervention in Secondary Type 2 Endoleaks

Whether to intervene or not and the exact point in time when to intervene for EL2 are a topic of ongoing debate [32–35]. EL2 are inherently low flow and are often transient, resolving following thrombosis of the aneurysm sac and reversal of anticoagulation. A recent meta-analysis of four major EVAR trials including 2783 patients showed an EL2 incidence of 11.7%, a reintervention rate of 22% for these EL2 (99 of 435 detected EL2); and no evidence that EL2, whether treated or not treated were associated with worse survival [36]. In fact the risk of aneurysm rupture in the presence of an isolated type 2 endoleak is exceptionally low [33]. The current consensus is that one should treat a persistent EL2 when they are associated with a significant sac size increase, commonly considered as at least 5 mm over 6 months [37]. In the absence of an enlarging sac size, patients with type 2 endoleak should be kept under follow-up imaging by CTA or US based on standard local protocols.

Embolisation is the main treatment for EL2. The aim of intervention is to obliterate the endoleak cavity, which is analogous to the central nidus in a vascular malformation. This is best achieved by occlusion of the supplying arteries (e.g. IMA) as well as the endoleak cavity. There are a variety of embolisation techniques depending on the anatomy of the artery supplying the endoleak and the available route to access the endoleaks.

- **Transarterial Embolisation**

**Fig. 2:**
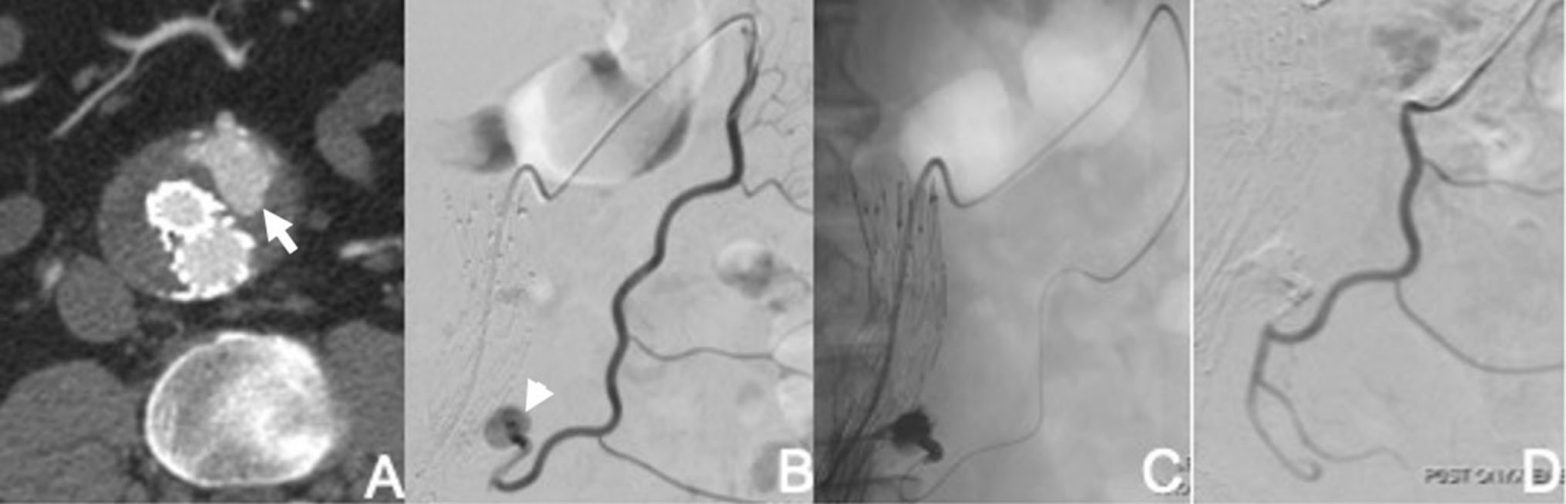
Transarterial embolisation of type 2 endoleak. **A** Axial CT image shows endoleak arising close to origin of IMA (arrow). **B** Angiogram via microcatheter placed into middle colic branch of SMA confirms endoleak (arrow head). **C** Microcatheter passed into endoleak cavity via the left colic branch of the IMA, and embolisation performed with Onyx. **D** Completion angiogram shows no further filling of endoleak

The most common technique is transarterial catheterisation of the dominant feeding vessel via communicating arteries supplying the vessel. This approach is performed under conscious sedation and requires an accessible route from an aortoiliac vessel, via collaterals to the vessel feeding the endoleak and ideally the endoleak cavity itself. The technical success of this approach is limited if the responsible feeding vessel cannot be cannulated or if a viable path to the endoleak cavity cannot be found. Transarterial embolisation is most successful for endoleaks originating from the IMA (Fig. 2). For embolisation of IMA endoleaks, a long 6-F sheath is inserted via a common femoral artery access and the tip placed at the SMA origin. A reverse-curve catheter (e.g. Simmons) is used to catheterise the SMA and an angiogram is performed: a. to confirm filling of the endoleak cavity via retrograde filling of the IMA and b. to depict the route to the IMA via the middle colic branch of the SMA and the left colic branch of the IMA. The sheath is advanced into the SMA to provide additional support. A hydrophilic 0.035 inch guidewire is advanced into the middle colic artery, followed by the selective catheter. A microcatheter is advanced coaxially through the middle colic branches of the SMA, into the left colic artery and subsequently into the IMA and the endoleak cavity. Embolisation is commonly performed using liquid embolics including glue and Onyx, although use of other agents such as gelfoam and thrombin have also been reported. The authors prefer using either a liquid embolic agent alone (Onyx or Glubran), or a combination of pushable coils and a liquid embolic.

**Fig. 3:**
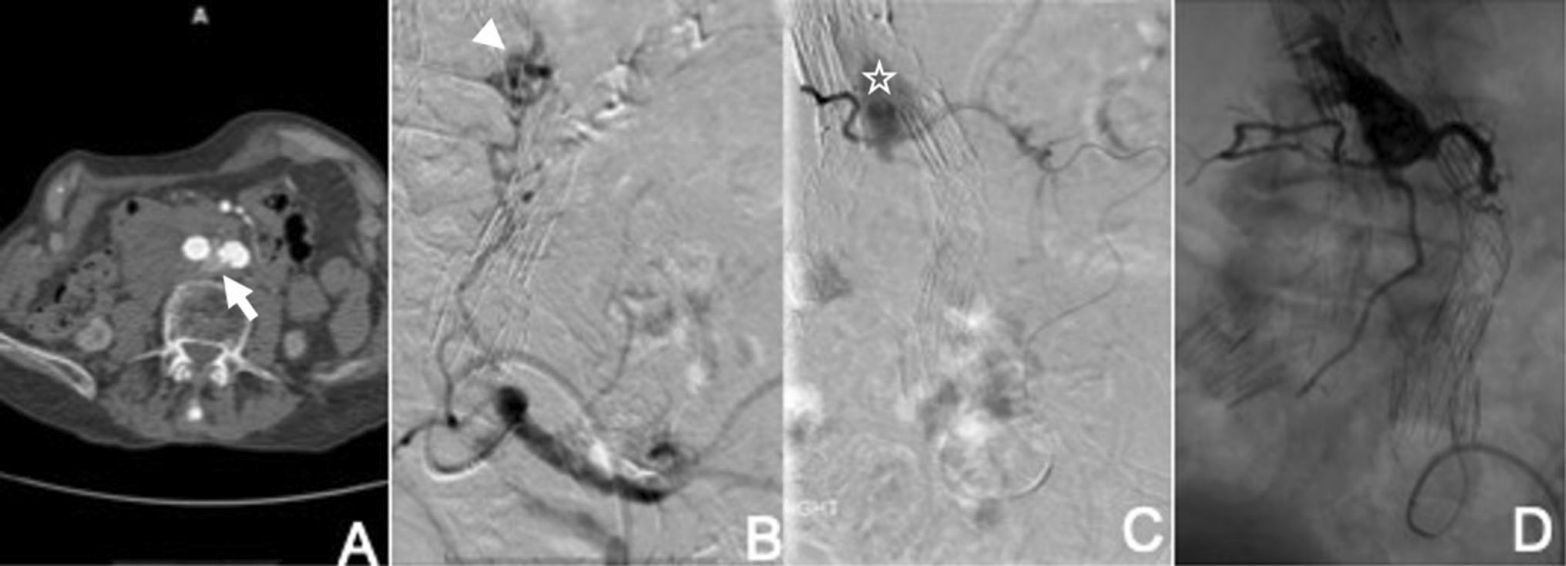
Transarterial embolisation of type 2 endoleak arising from iliolumbar artery. **A** Axial CT image shows endoleak arising from a left lumbar artery (arrow). **B** Angiogram following catheterisation of the left internal iliac artery shows filling of the endoleak cavity (nidus) by the left lumbar artery (arrowhead). **C** Angiogram following selective microcatheter catheterisation of the endoleak cavity through the tortuous iliolumbar artery shows endoleak cavity (asterix) and several exit vessels. **D** Complete embolisation of endoleak cavity and exiting branches using Onyx

Type 2 endoleaks arising from iliolumbar arteries can be treated in a similar manner (Fig. 3). The main difference to the above is the use of a short 6-F sheath placed in the ipsilateral femoral artery, using an appropriate catheter to catheterise the internal iliac artery (e.g. Sos Omni) and advancing a microcatheter coaxially via the ascending iliolumbar artery into the feeding lumbar artery or ideally the endoleak cavity. A steerable sheath may be helpful in some cases. This approach is more challenging with a lower success rate compared to IMA endoleak embolisation, because of the difficulty cannulating the responsible lumbar feeding vessel through the frequently tortuous and small caliber arterio-arterial communications.

In these cases, if the endoleak cavity cannot be accessed, it may be possible to successfully embolise the endoleak by injecting a low viscosity Onyx preparation (Onyx 18) from a more proximal location, which may flow gradually into the endoleak cavity [38]. However, proximal embolisation of the iliolumbar artery itself without occlusion of the endoleak cavity may result in recurrence from other collateral vessels that may supply the endoleak cavity.

Outcomes of transarterial embolisation: There is a relatively wide spread of reported technical and clinical success rates for this technique (Table 3), which is reflected in several meta-analysis and systemic reviews published recently [36, 37, 39]. One of the largest single cohorts published in 2012 of 95 patients undergoing 140 embolisation procedures (predominantly transarterial but a few other techniques too), using a range of embolics (glue 61%, coils 29%, glue and coils 7% and Gelfoam 3%) showed 81.5% freedom from aneurysm sac expansion at one year but a significant decrease to 43.7% at five years, with an associated increase in the number of repeat embolisation procedures required [40]. The high long-term failure rate may in part reflect failure to completely occlude the endoleak cavity due to shortcomings of earlier techniques such as embolising the feeding vessel but not the endoleak cavity and performing embolisation using coils alone without liquid embolic.

**Table 3:** Main publications on outcomes of EL2 transarterial embolisation, published since 2009

Authors	No. of endoleak cases	Patient population	Embolic material	Technical success (%)	Follow-up length mean–months (range)	Clinical success (%)	Additional comments
Ribe et al. [79]	18	600	Onyx	18 (100)	19 (3–60)	16 (89)	EL2 source: IIA in 7, IMA in 7 and lumbar artery in 4 cases
Wojtaszek et al. [80]	22	22	Onyx	17 (77)	17 (3–38)	17/21 (81)	
Hongo et al. [81]	20	20	NBCA and coils	18 (90)	18.5 (6–36)	13 (65)	
Müller-Wille et al. [82]	11	11	Onyx	6 (55)	26 (6–50)	8 (73)	Clinical success defined as no increase in sac size on follow-up imaging
Funaki et al. [83]	16	25	Cyanoacrylate, coils and ethylene vinyl alcohol copolymer	14 (88)	27.5 (6–88)	16 (100)	Clinical success defined as no increase in sac size on follow-up imaging

Internal iliac artery (IIA), Inferior mesenteric artery (IMA)

There is no consensus on the optimal embolic agent or combination of embolic agents for transarterial embolisation.

Where conventional transarterial embolisation is not possible or fails, other techniques may achieve access to the endoleak cavity for embolisation. These include transiliac paraendograft embolisation (TIPE), direct sac puncture and transcaval embolisation.

- **Transiliac Paraendograft Embolisation (TIPE)**

TIPE is a novel technique for treating type 2 endoleaks that cannot be accessed by the standard transarterial route and involves passing a catheter and hydrophilic wire into the potential space between the iliac limb endograft and the vessel wall. Once access into the paraendograft space is obtained, the catheter and wire are advanced superiorly using standard catheter–guidewire manipulation techniques between the graft and the artery wall until access to the sac thrombus is achieved. Further catheter–guidewire manipulation within the sac thrombus may enable the interventionist to access the endoleak nidus itself, which is heralded by blood flow from the catheter. After performing an endoleakogram to define the anatomy of the endoleak, the nidus and any visible and accessible feeding vessels are embolised with a liquid embolic and coils or a combination of these agents. This technique can be performed during the same procedure as a failed attempt at conventional transarterial embolisation.

Using this technique, Coppi and colleagues reported successful embolisation of the sac in 16 of 17 patients [41] using a 9F sheath, with one adverse event of a procedural type Ib endoleak. In the authors experience, paraendograft access with a 4/5Fr catheter alone or a 6Fr sheath is technically adequate and minimises the risk of a procedural type Ib endoleak [42]. In practice, procedural success is limited by difficulty in accessing the paraendograft space and accessing the endoleak nidus even when the sac thrombus has been accessed. Embolisation of the sac thrombus if the nidus cannot be accessed is of no benefit.

- **Direct Sac Puncture**

**Fig. 4:**
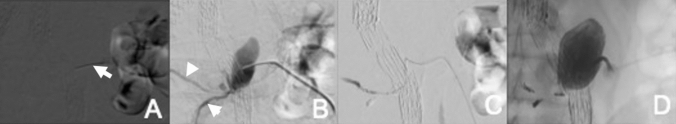
Direct sac puncture and embolisation of type 2 endoleak. **A** Fluoroscopic guided access into the endoleak cavity via 18G Chiba needle (arrow), using bony landmarks and aortic endograft markers. **B** Angiogram via 18G needle confirms endoleak cavity (nidus) and several exit vessels (arrow heads). **C** Embolisation of exit vessels with micro-coils via microcatheter. **D** Subsequent embolisation of the endoleak cavity with Onyx

This involves the direct percutaneous puncture of the aneurysm sac. It is most commonly performed via a translumbar approach with the patient positioned prone on the operating table but may also be performed transabdominally [56] when there is an anterior endoleak. It can be performed under general anaesthesia, or under sedation and local anaesthesia, depending on the patient and the potential difficulty of the procedure. Prior CT imaging is initially reviewed to assess the approach to the endoleak cavity. Access is obtained using fluoroscopic guidance or targeted C-arm CT software available on most modern angiographic equipment. An 18 or 20G coaxial needle is advanced until there is brisk, pulsatile blood flow through the needle, indicating a satisfactory position within the endoleak cavity. An angiogram is performed to depict the anatomy of the endoleak and to plan the subsequent embolisation. At this point, the needle is exchanged for a 4,5 or 6Fr sheath over a stiff guidewire wire and a short selective catheter (e.g. KMP, Bolia, Cobra) is advanced into the endoleak cavity. With the catheter tip located in a stable position in the endoleak nidus, a microcatheter is advanced into the endoleak. If feeding vessels are visualised and can be catheterised, these should be embolised first with coils, a liquid embolic or small plugs. It may not be possible to access any feeding arteries, and strenuous and lengthy efforts should not be made to do this as embolisation of the nidus is the main aim of this procedure. After embolisation of any feeding arteries, the nidus is embolised with a liquid embolic, coils or a combination (Fig. 4).

Outcomes of Direct Sac Puncture Embolisation: There are only two papers that have specifically reported the outcomes of direct sac puncture embolisation [56, 57]. In the larger of these studies, Zener et al. (2018) reported on 33 transabdominal embolisations in 30 patients using a range of embolic agents with a technical success rate of 97% and clinical success of 85%, defined as freedom from sac growth (Table 4).

**Table 4:** Main publications directly assessing outcomes of direct sac puncture and embolisation for EL2

Authors	No. of endoleak cases	Patient population	Embolic material	Technical success (%)	Follow-up length mean–months (range)	Clinical success (%)	Transabdominal or Translumbar
Zener et al. [56]	33	30	Glue only (45.5%), glue/coils (36.4%) and Onyx with or without glue/coils (18.1%)	29 (97)	15	23/27 (85)	All transabdominal
Carrafiello et al. [57]	8	8	Thrombin only in 5 cases, thrombin and glue in 2 cases and Onyx in 1 case	8 (100)	36 (24–46)	8 (100)	All translumbar

There are several papers that report outcomes comparing direct sac puncture and transarterial embolisation [58–62]. Recently a systematic review of 32 studies comprising 393 interventions for type II endoleak, compared outcomes for translumbar embolisation and transarterial embolisation. The review reported that translumbar embolisation had a higher technical success rate (81% vs. 63%), fewer cases of endoleak recurrence (19% vs 36%) and a lower complication rate (0% vs 9%) when compared with transarterial embolisation [63]. However, this review includes data from a heterogenous cohort of studies using a variety of techniques and embolic agents conducted retrospectively, and its overall conclusion that direct sac puncture is more effective than transarterial embolisation remains open to question. Clearly, one must remember that many interventionists select the embolisation method on a case-by-case basis dependent on the anatomy of the endoleak and their perception regarding which technique is likely to be more likely to be successful. Therefore, in the absence of evidence from randomised studies comparing techniques, the reviewer must bear this in mind. It is the author’s opinion, that if an expert interventionist in all methods selects the specific technique based on the vascular anatomy, then the outcomes of a technically complete embolisation should be comparable, whichever technique is used.

- **Transcaval Embolisation**

In this technique, transcaval access into the endoleak cavity is achieved by using an angled-tip catheter and an angled sheathed needle (e.g. TIPSS set) to penetrate the IVC wall and enter the endoleak cavity. There is limited data on this technique, which is summarised in Table 5. The largest cohort included 29 patients, reported by Giles et al. [86], with technical success achieved in 90% and no significant adverse events, although 5 patients required reintervention.

**Table 5:** Main publications directly assessing outcomes of transcaval embolisation for EL2

Authors	No. of endoleak cases	Patient population	Embolic material	Technical success (%)	Follow-up length mean–months (range)	Clinical success (%)	Additional comments
Scali et al. [84]	6	6	Coils + thrombin	6 (100)	8.1 (2–22)	4 (66)	Thrombin used in 2 IV ultrasound in 4 and intraoperative CT in 2 cases
Gandini et al. [85]	29	26	Coils + glue/thrombin	9 (100)	25 (14–31)	25 (86)	Feeding artery also embolised in 20 cases, all with no recurrence
Giles et al. [86]	29	29	Coils + thrombin	24 (83)	16.5 (± 10.4)	20 (70)	Thrombin injection used in 5, IV ultrasound in 4 and intra operative CT in 5 cases
Mansueto [87]	12	12	Coils + thrombin	11 (92)	12	10 (83)	Thrombin injection used in 5, IV ultrasound in 4 and intra operative CT in 5 cases

- **Surgery**

Surgical options include laparoscopic clipping of the lumbar or inferior mesenteric arteries, surgical fixation of the endograft to the aortic wall or open aneurysmectomy. These are treatments of last resort for cases where the above techniques have been unsuccessful or not feasible. In view of the increasing variety of embolisation techniques available, surgical intervention is seldom required.

### Selecting the Best Approach to Manage Type 2 Endoleaks

**Fig. 5:**
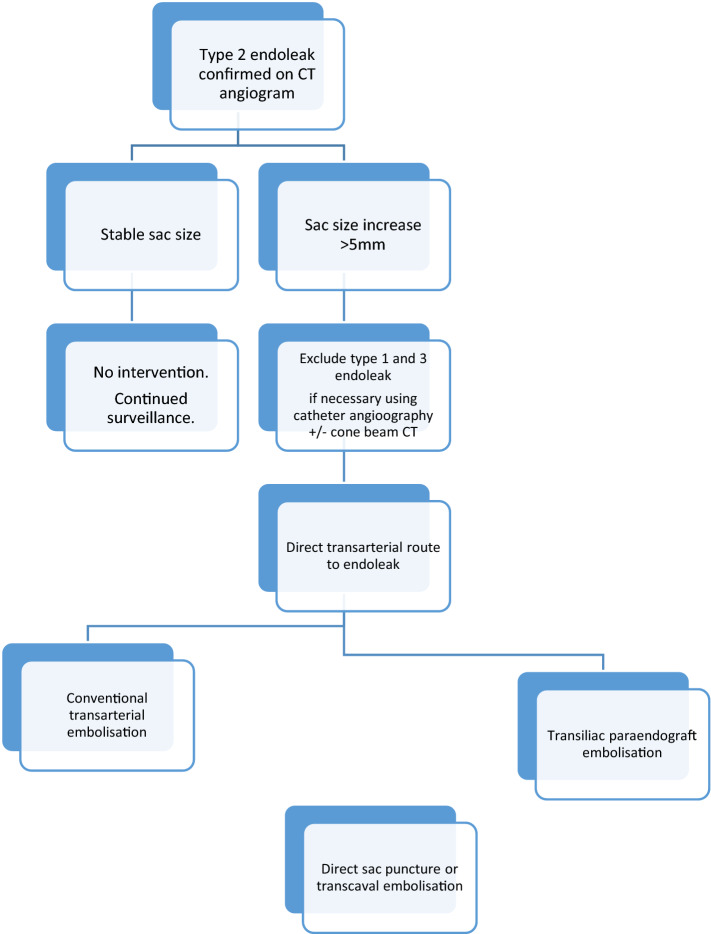
Summary of author’s approach to management of type 2 endoleaks

As described, there is a range of embolisation techniques that may be utilised for EL2. In some cases, more than one technique may be undertaken to achieve embolisation. Figure 5 illustrates a summary of the author’s practice in managing EL2.

In principle, when faced with a suspected EL2 and increasing sac size, it is imperative to consider the possibility of an occult type 1 or type 3 endoleak disguised as a type 2 endoleak, where opacified aortic side branches are acting as exit vessels rather entrance vessels. CT imaging is usually sufficient for this, but if CTA is inconclusive, catheter aortography (sometimes combined with cone-beam CT) can be used to help confirm the source of the endoleak and to plan treatment. Contrast-enhanced ultrasound enables real-time imaging of arterial flow into the endoleak and can be useful as a problem solving tool, particularly for assessment of the type and anatomy of endoleaks.

A catheter angiogram with a view to direct transarterial embolisation is usually the first intervention. If the endoleak is arising from retrograde flow in the IMA, a direct coaxial microcatheter catheterisation via the SMA is usually feasible. Endoleaks arising from a lumbar or iliolumbar branch are often less amenable to transarterial embolisation than IMA embolisation. Nevertheless, transarterial embolisation of a lumbar EL2 should be still attempted at the time of the diagnostic catheter angiogram. If transarterial embolisation is unsuccessful, a transiliac paraendograft catheterisation of the endoleak sac can be attempted during the same sitting. If these are unsuccessful, consideration should be given to utilising the alternative access routes of percutaneous direct sac puncture or the transcaval route. It is the author’s preference to schedule these at a later date as a separate procedure.

3. **Type 3 Endoleaks**

Type 3 endoleaks result from a structural defect of the endograft, and can be subdivided into EL 3a, caused by component modular disconnection and EL 3b, secondary to a fabric tear. They are relatively uncommon, and increasingly so with modern stent graft designs. A recent retrospective study of 967 EVAR cases reported type 3 endoleaks in 12.7% for first and second generation endografts and 1.3% in third generation endografts [64]. These are high flow endoleaks similar to type 1 endoleaks, resulting in sac pressurisation and therefore EL3 mandate immediate treatment. The standard treatment is relining the endograft by deploying a new endograft within the preexisting graft. There are a few isolated case reports of embolisation of EL3 where relining is not possible or fails.

4. **Type 5 Endoleaks**

These are also termed ‘endotension’ and are defined as an increase in sac size in the absence of an identifiable endoleak. When assessing potential cases, catheter angiography together with cone beam CT may be useful in excluding the presence of an endoleak or another cause for sac expansion. If no cause is found, observation may be a valid option for some of these cases, as these endoleaks are not directly associated with high pressure, but the criteria for conservative management are unclear [36]. Options for intervention in cases of increasing aneurysm sac size include the use of extension cuffs, relining the endograft and conversion to open repair [65].

## Management of Secondary Endoleaks—TEVAR

Thoracic aortic aneurysms (TAAs) affect 10.4 in 100,000 people per year, with an estimated incidence of rupture and dissection at 3.5 per 100,000 per year, with a high (90%) mortality rate in cases of acute rupture [66, 67] The aim of TEVAR is to reduce these risks and many studies have confirmed favourable long-term outcomes after endovascular repair. Overall, all types of endoleaks after TEVAR range occur in 9.5 to 15.8% of procedures, and there are limited data regarding the incidence of secondary vs primary endoleaks. Below we provide an overview of the management of endoleaks following TEVAR for aneurysms and do not address those performed for aortic dissections.

**• **Type 1a Endoleaks

Type 1 endoleak occurs in 3 to 16% of cases [68–71]. The treatment options are mostly endovascular with a low rate to open conversion of 3.6% [72]. Secondary proximal type 1 endoleaks are due to an ongoing poor seal at the proximal attachment site, dilatation of the proximal attachment site or distal endograft migration. Management of secondary EL1a is primarily by extension proximally of endograft coverage by additional endografts to achieve a seal. There are a few reports of the use of EndoAnchors to treat proximal type 1 endoleaks [73, 74]. However, in general, proximal endograft coverage is the optimal treatment method. If the proximal landing zone is close to or involves the aortic arch arteries, efforts should be made to preserve flow into these arteries by surgical debranching, fenestrations, branches or chimneys. A comprehensive discussion of these advanced techniques is provided in the article entitled "Various endoluminal approaches available for treating pathologies of the aortic arch".

There are a few reports of the use of embolisation to treat EL1a where other techniques are not feasible and consideration to this option should be given if the requisite interventional skills are available for this highly challenging treatment option. Although the published data are limited to case reports, the procedural outcomes have been satisfactory. Day et al. reported two cases of successful EL1a embolisation post-TEVAR and with no recurrence at the 12 months follow-up [75]. The technique involves common femoral artery access, the use of a long sheath to access the proximal aorta, and a reverse curve-shaped catheter to engage the endoleak cavity.

Depending on the anatomy of the endoleak, detachable coils, a liquid embolic agent (e.g. Onyx) or a combination can be used to occlude the endoleak; however, the risk of embolisation to the cerebral arteries, especially when using liquid embolics should be considered. In certain cases, if the endoleak cannot be accessed from the aortic lumen, direct percutaneous access [76, 77] can be used. Patients who cannot be treated by endovascular or surgical methods are managed conservatively, with the risk of rupture that this entails.

- **Type 1b Endoleaks**

**Fig. 6:**
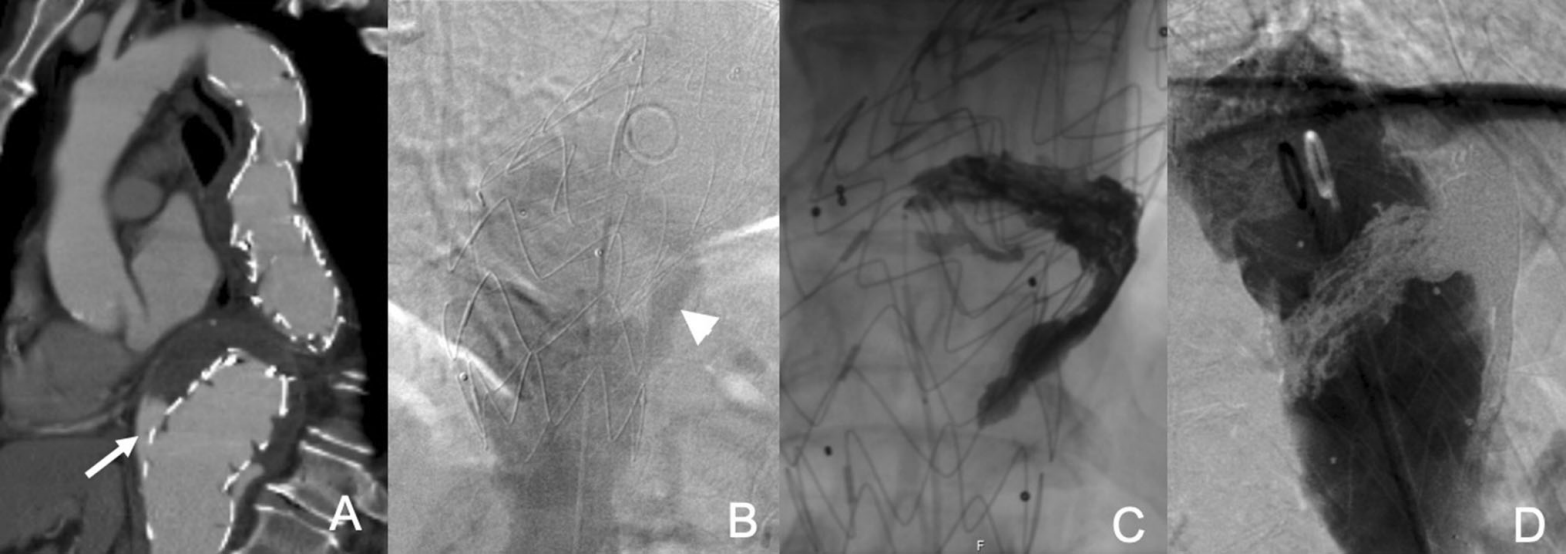
EL1b embolisation following TEVAR. **A** Sagittal CT angiogram shows distal EL1 in patient with a thoracic endograft (arrow). **B** Aortic angiograms confirm EL1b (arrow head). **C** Embolisation of the endoleak cavity with via a microcatheter. **D** Final angiogram shows successful endoleak embolisation

Similarly to EL1a, EL1b are treated by distal endograft extension. If this involves extending across the upper abdominal visceral arteries, this can be achieved in association with surgical debranching (hybrid procedure), FEVAR, BEVAR and ChEVAR. There are a few reports of embolisation of EL1b after TEVAR, and an example is shown in Fig. 6.

Patients with chronic dissection who develop late false lumen expansion and require endograft extension distally can also be treated in this way, although there are also options for endovascular occlusion of the false lumen using techniques such as the Candy-Plug procedure, Knickerbocker procedure, and embolisation of the false lumen with coils and liquid embolics. Refer to the article entitled "Role of endoluminal techniques in the management of chronic type B aortic dissection" in this special issue.

- **Type 2 Endoleaks**

Type 2 endoleaks after TEVAR result from retrograde flow into the aneurysm sac from branches of the thoracic aorta, but are less common compared to EVAR, with a reported incidence of 3.3% in the EUROSTAR Registry [78]. The most common cause of an EL2 post-TEVAR is retrograde flow from the left subclavian artery in patients where stent grafts have been placed across the origin of the left subclavian artery. If there is an increase in sac size, the proximal subclavian artery should be embolised with a plug or coils from the ipsilateral brachial or radial artery access, taking care to avoid the origin of the left vertebral artery, so that perfusion to the left arm via the left vertebral artery is preserved.

Type 2 endoleaks may also arise from other branches of the thoracic aorta such as the intercostal and bronchial arteries. These are usually managed conservatively, unless there is an increase in the sac size that can only be attributed to the EL2 [72]. Although challenging to treat, embolisation of these EL2 may be feasible by the transarterial or percutaneous direct sac puncture route, although reports of the efficacy of these techniques are very limited.

## Conclusion

Secondary endoleaks remain an ongoing challenge following endovascular repair of the thoracic and abdominal aorta, mandating a significant burden for healthcare providers and patients in terms of surveillance and reintervention. Type 1 and 3 endoleaks result from direct communication between the high-pressure intraluminal flow in the aortoiliac vessels and the aortic sac. The significance of these is well understood, requiring prompt treatment, which includes endovascular and surgical options. Type 2 endoleak management remains a subject of debate however, with embolisation as the mainstay treatment reserved for persistent cases with a significant sac size increase.
